# The effect of acupuncture on exercise capacity in patients with COPD is mediated by improvements of dyspnea and leg fatigue: a causal mediation analysis using data from a randomized controlled trial

**DOI:** 10.1186/s12906-024-04353-0

**Published:** 2024-01-20

**Authors:** Takumi Kayo, Masao Suzuki, Tadamichi Mitsuma, Fumihiko Fukuda

**Affiliations:** 1 Department of Acupuncture and Moxibustion, Faculty of Acupuncture and Moxibustion, Meiji University of Integrative Medicine, Nantan, Kyoto Japan; 2 Department of Kampo Medical Research Institute, Aizu Medical Center, Fukushima Medical University, Aizuwakamatsu, Fukushima Japan

**Keywords:** Chronic obstructive pulmonary disease, Exercise capacity, Dyspnea, Leg fatigue, Acupuncture, Mediation analysis

## Abstract

**Background:**

Acupuncture is known to improve exercise capacity in patients with chronic obstructive pulmonary disease (COPD), but its mechanism remains unknown. Whether acupuncture improves exercise capacity in patients with COPD through alleviation of leg fatigue and dyspnea is examined by applying causal mediation analysis to previous trial data.

**Methods:**

Sixty-two patients with COPD completed treatments with either real or placebo acupuncture once a week for 12 weeks. Walk distance measured using the 6-minute walk test and intensities of leg fatigue and dyspnea in the modified Borg scale were evaluated at baseline and after treatment. The intervention effect of acupuncture against the placebo acupuncture on two mediators, changes in leg fatigue and dyspnea, and whether they mediated improvements in walk distance, were analyzed.

**Results:**

Linear regression analysis showed that the unstandardized regression coefficients [95% confidence interval (CI)] for the intervention effect by acupuncture were -4.9 (-5.8–-4.0) in leg fatigue and -3.6 (-4.3–-2.9) in dyspnea. Mediation analysis showed that when changes in leg fatigue were considered as a mediator, direct effect, indirect effect and proportion mediated were 47.1 m (95% CI, 4.6–85.1), 34.3 m (-2.1–82.1), and 42.1%, respectively, and when changes in dyspnea were considered as a mediator, they were 9.8 m (-32.9–49.9), 72.5 m (31.3–121.0), and 88.1%, respectively, and the effects of joint mediator were -5.8 m (-55.4–43.9), 88.9 m (32.7–148.5), and 107.0%, respectively.

**Conclusion:**

The improvement in exercise capacity by acupuncture is explained by changes in both leg fatigue and dyspnea.

## Introduction

Patients with chronic obstructive pulmonary disease (COPD) are affected by multiple factors, such as ventilatory limitation, gas exchange limitation, cardiac limitation, lower limb muscle dysfunction and respiratory muscle dysfunction, and their exercise is restrained mainly due to worsened leg fatigue and dyspnea during exertion [1–4]. This exercise intolerance is a predictor of mortality and exacerbation-related hospitalization for patients with COPD [5, 6], and is also associated with quality of life and physical activity [7, 8]; therefore, improvement regarding exercise intolerance is an important therapeutic goal [9].

Pharmacological therapies such as bronchodilators and pulmonary rehabilitation can reduce symptoms in patients with COPD, as well as improve their exercise capacity. However, according to the longitudinal analysis of the data from the Swedish National Register of COPD, 74% of the patients who inhaled triple therapy of long-acting muscarinic antagonists, long-acting beta-2-agonists and inhalation corticosteroids along with physiotherapy had persistent dyspnea that interfered with activities with a modified medical research council score of 2 or higher, which indicates that the current standard treatment alone is not sufficient [10].

On the other hand, reports of acupuncture use for COPD have been increasing in recent years. Our previous randomized controlled trials showed that acupuncture improved exercise capacity in patients with COPD, and other clinical trials have subsequently reported the same effect [11–13]. However, it is not well understood how acupuncture improves exercise capacity in patients with COPD. Since the majority of patients with COPD stop exercising due to dyspnea and leg fatigue [2–4], the effects of acupuncture may have been achieved through alleviation of these symptoms. However, no studies to date have investigated this. Therefore, by applying causal mediation analysis to the existing clinical trial data [11], we investigated how much of the improvement in exercise capacity for patients with COPD induced by acupuncture can be explained by its alleviating effects in leg fatigue and dyspnea.

## Methods

### Study design and patients

This study was a secondary analysis of data from previous randomized, single-blind, placebo-controlled, and parallel group trial to see the effects of acupuncture on patients with COPD. Details of the trial design have previously been published [11], so we will describe them briefly. The trial was conducted in the Kansai region of Japan between July 2006 and March 2009. Sixty-eight patients who were diagnosed as having stage II, III, or IV COPD, in accordance with the definition and criteria of the Global Initiative for Chronic Obstructive Lung Disease (GOLD) guidelines, were assigned to the real acupuncture group (*n* = 34) or the placebo group (*n* = 34). The trial was conducted in accordance with the Declaration of Helsinki and its amendments, as well as the Guidelines for Good Clinical Practice for Epidemiological Studies and Clinical Research issued by the Japanese Ministry of Health, Labour and Welfare, and the research protocol is registered in the UMIN Clinical Trials Registry (UMIN000001277). In the previous trial, written informed consent was obtained from all participants. This study used only the data collected from this previous trial. Therefore, we disclosed information about this study on our website and provided the participants with the chance to opt-out. The Research Ethics Committee of Meiji University of Integrative Medicine approved this study (Approval number 2021-016).

This study included 62 participants who completed the protocol for each treatment group and had a complete dataset of mediators and outcomes at baseline and follow-up.

### Intervention

Patients in the real acupuncture group received acupuncture treatment once a week for 12 weeks in addition to daily medication. The standardized acupuncture points used in the previous study were: Zhongfu (LU 1) and Taiyuan (LU 9) in the lung meridian; Futu (LI 18) in the large intestine meridian; Guanyuan (CV 4) and Zhongwan (CV 12) in the conception vessel; Zusanli in the stomach meridian (ST 36); Taixi (KI 3) in the kidney meridian; Wangu in the gallbladder meridian (GB12); and Feishu (BL 13), Pishu (BL 20), and Shenshu (BL 23) in the bladder meridian [11]. These acupuncture points were selected based on the past research on acupuncture for pulmonary dysfunction [14, 15] and the literature describing traditional acupuncture point prescriptions for bronchial asthma and chronic bronchitis. And some other acupuncture points (such as Futu [LI18] and Wangu [GB12]) which are on the accessory muscles of respiration were added. Treatment frequency was determined based on our previous matched-pair study showing the efficacy of once-weekly acupuncture [15]. For the placebo group, placebo acupuncture needles, Park sham devices, which have a blunt needle tip and can be retracted while still appearing to pierce the skin, were used [16]. The real and placebo needles appear similar and are the same size (0.35 mm × 70 mm, stainless steel, Dong Bang Acupuncture Inc., Bundang, Seongnam, Korea).

For the real acupuncture group, needles were inserted to a depth ranging from 5 to 25 mm and manually rotated clockwise and counter-clockwise for 3–4 min at each point during 50-min treatment periods. No electrical stimulation was performed. Perception of de qi (tingling, numbness, heaviness, and other feelings that occur after an acupuncture needle is inserted) during insertion and/or manipulation was confirmed at each point.

The placebo group underwent treatment at the same acupuncture points and frequency as the real acupuncture group. Perception of sensation during treatment sessions in the placebo group included pricking or poking.

Patients in both groups were informed in advance that both real and placebo acupuncture needles could cause some sensations. The acupuncturist asked patients if they felt any sensations, which could be considered “de qi”, during the treatment, but they were not informed that such sensations were peculiar to real acupuncture. Evaluation of blinding at the end of the trial revealed that the blinding was successful and patients were unable to distinguish between real and placebo acupuncture [11, 17].

### Outcome and mediators

A 6-minute walk test was performed at baseline and postintervention, and changes in walk distance in meters from baseline were evaluated as outcomes [18]. Immediately after the walk test, the intensities of leg fatigue and dyspnea were measured by the modified 10-point Borg category ratio scale and the changes from baseline were evaluated as mediators [19].

**Fig. 1:**
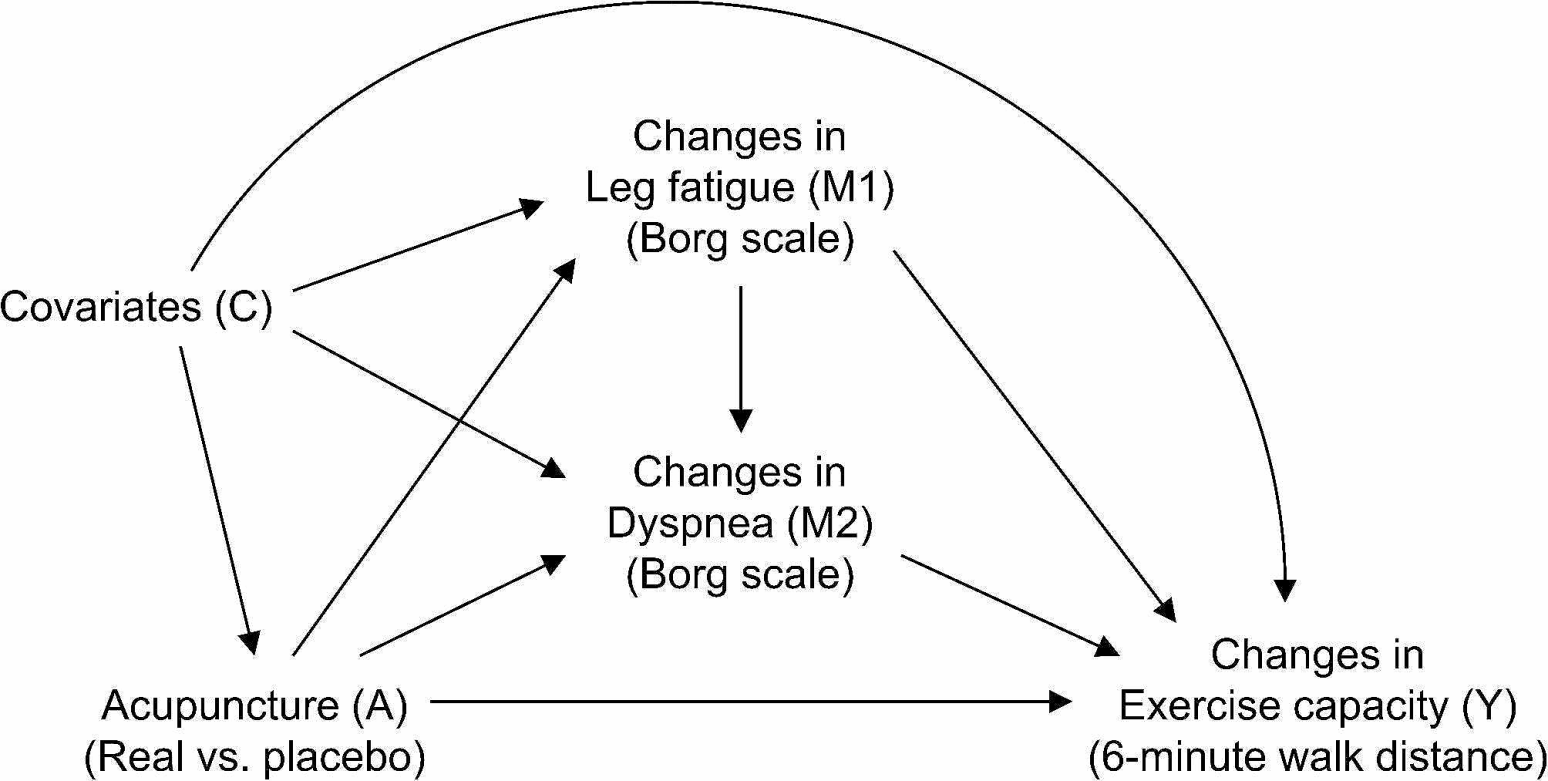
Assumed causal graph The relationships between intervention (A), mediators (M1 and M2), outcome (Y), and covariates (C) that were assumed in this study are shown

We hypothesized the relationship between intervention (A), two mediators (M1, M2), outcome (Y), and covariates (C) as shown in Fig. 1. This hypothesis was based on the following reported findings: exercise capacity in patients with COPD is mainly limited by leg fatigue and dyspnea [2–4]; experimentally induced leg fatigue in healthy subjects exacerbates dyspnea on exertion [20, 21]; inhibition of sensory muscle afferents in legs by spinal anesthesia improves dyspnea during exercise and exercise capacity inpatients with COPD [22]; acupuncture is likely to improve muscle fatigue [23]; acupuncture clinical trials for patients with COPD observed improvement in exercise capacity as well as improvement in dyspnea on exertion [11–13]. Therefore, we considered the possible mechanism of acupuncture to improve exercise capacity in patients with COPD by hypothesizing the following pathways; one affecting leg fatigue but not affecting dyspnea (path A-M1-Y in Fig. 1), one first affecting leg fatigue and subsequently affecting dyspnea (path A-M1-M2-Y), one directly affecting dyspnea (path A-M2-Y), and one which does not belong to any of the above (path A-Y).

### Covariates

The measured values of the 6-minute walk distance, leg fatigue, and dyspnea at baseline were considered covariates in mediation analysis. For sensitivity analysis, we added age and baseline values of body mass index (kg/m2) and inspiratory capacity (L) to the covariates. These were selected since they are variables that could affect both outcome and mediators [6, 24–27].

### Statistical analysis

For the baseline characteristic values, mean ± standard deviation was calculated for the continuous variables, and frequency was calculated for the categorical variables. To assess the intervention effect of acupuncture against the placebo acupuncture on each of the two mediators, we fitted a linear regression model conditional on the baseline values and intervention groups respectively, and then estimated the unstandardized regression coefficients with 95% Confidence Intervals (CI).

Causal mediation analysis was performed to decompose the total effect (TE) of acupuncture against the placebo acupuncture on the 6-minute walk distance into the natural direct effect (NDE) and the natural indirect effect (NIE) [28]. NDE is, to put it simply, the effect of intervention on the outcome that works through non-mediator mechanisms, and NIE is the effect through a mediator. We performed mediation analyses when leg fatigue, dyspnea, and their joints were considered mediators, respectively. By doing these analyses, we were able to assess the extent to which the effects of acupuncture were explained by each mediator. In addition, by comparing the mediation effects (i.e., NIE) when leg fatigue was considered as a mediator and when leg fatigue and dyspnea were considered jointly, we were able to assess the magnitude of the effect of the pathway that directly affected dyspnea not through leg fatigue (path A-M2-Y in Fig. 1). Furthermore, by comparing the sum of the individual NIE of leg fatigue and dyspnea with the joint NIE, we were able to assess the possibility that mediators affect one another. We performed the mediation analysis using a simulation-based approach using the “Direct Counterfactual Imputation Estimation” implemented in the R package “CMAverse” [29, 30]. This approach requires regression models for the mediator and for the outcome. First, we estimated NDE, NIE, TE, and the proportion mediated (PM = NIE divided by TE) when the changes in leg fatigue (M1) were considered as the mediator. We fitted a linear regression model for the M1 conditional on the intervention group (A) and covariates (C), which included baseline values of 6-minute walk distance, leg fatigue, and dyspnea, and a linear regression model for the outcome (Y) conditional on A, M1, the product term of A and M1, and C. Next, we estimated NDE, NIE, TE, and PM when the changes in dyspnea (M2) were considered as the mediator. We estimated a linear regression model for the M2 conditional on A and C, and a linear regression model for the Y conditional on A, M2, the product term of A and M2, and C. Thirdly, we estimated the NDE, NIE, TE, and PM when M1 and M2 were considered joint mediators [31]. We used the same linear regression models previously estimated for M1 and M2, and estimated a linear regression model for the Y conditional on A, M1, M2, the product term of A and M1, the product term of A and M2, and C.

We performed two sensitivity analyses. First, in order to evaluate the robustness to the addition of covariates, the simulation-based approach was performed again after adding age and the baseline values of body mass index and inspiratory capacity to covariates. Second, a weighting approach was performed to evaluate the robustness to the different approaches for effect estimation [31]. In order to implement the weighting approach, outcome and exposure models are required. So, for the outcome model, we fitted a regression model similar to the simulation-based approach, and for the exposure model we fitted a logistic regression model for intervention A conditional on C. The weighting approach was carried out using “The Weighting-based Approach” implemented in the R package “CMAverse” [30]. The 95% CIs for each effect in all mediation analyses were obtained using 1,000 bootstraps. All analyses were performed using R software (version 4.1.1).

## Results

### Participants

Of the 111 people who met the inclusion and exclusion criteria, 68 participated in the trial, and they were randomly assigned to a real or placebo acupuncture group with 34 each. Finally, 62 participants (30 in the real acupuncture group, 32 in the placebo group) who completed the protocol and had a complete dataset of outcomes and mediators at baseline and follow-up were included. The baseline characteristics of the subjects in each group are shown in Table 1. Information about the medications used on the subjects has been provided in the previous study [11]. All medications remained unchanged throughout the study period.

**Table 1:** Baseline characteristics of study participants

	Placebo (*n* = 32)	Real (*n* = 30)
Sex		
Male	30	27
Female	2	3
Age, mean (SD), years	71.9 (7.0)	72.1 (7.0)
Body mass index, mean (SD), kg/m^2^	21.3 (3.9)	21.7 (3.9)
Modified MRC dyspnea scale, mean (SD)	1.9 (1.2)	2.2 (1.0)
Brinkman index, mean (SD)^a^	1461.0 (775.7)	1258.2 (512.4)
GOLD criteria		
II	13	6
III	7	13
IV	12	11
Home oxygen therapy	10	8
6MWT		
6MWD, mean (SD), m	405.2 (111.2)	373.2 (115.2)
Leg fatigue, Borg scale, mean (SD)	5.1 (1.9)	5.7 (3.3)
Dyspnea, Borg scale, mean (SD)	4.2 (2.7)	5.5 (2.8)
SpO2, mean (SD), % lowest value	88.3 (6.1)	86.0 (7.0)
Pulmonary function, mean (SD)		
VC, L	3.15 (0.67)	2.88 (0.52)
VC, % predicted	100.3 (19.4)	94.6 (14.1)
IC, L	1.91 (0.50)	1.75 (0.37)
ERV, L	1.25 (0.32)	1.13 (0.38)
FVC, L	2.99 (0.67)	2.79 (0.55)
FEV1, L	1.15 (0.41)	1.04 (0.30)
FEV1, % predicted	47.9 (16.7)	46.0 (16.6)
FEV1/FVC, %	38.1 (11.3)	37.8 (11.8)

Placebo, placebo acupuncture group; Real, real acupuncture group; SD, standard deviation; MRC, Medical Research Council; GOLD, Global Initiative for Chronic Obstructive Lung Disease; 6MWT, 6-minute walk test; 6MWD, 6-minute walk distance; SpO2, oxyhemoglobin saturation as measured by pulse oximetry; VC, vital capacity; IC, inspiratory capacity; ERV, expiratory reserve volume; FVC, forced vital capacity; FEV1, forced expiratory volume in 1 s

^a^The Brinkman Index is calculated by multiplying the number of cigarettes smoked per day by the number of years smoked

### Effects on leg fatigue and dyspnea

Acupuncture yielded a clear therapeutic effect compared to the placebo for both leg fatigue and dyspnea (Table 2). As a result of the linear regression analysis, the unstandardized regression coefficient (95% CI) of the real acupuncture group compared with that of the placebo group was -4.8 (-5.8–-3.9) for leg fatigue and -3.6 (-4.3–-2.9) for dyspnea.

Changes in variables other than those analyzed in this study have been reported in previous studies [11]. Variables measured before and after the test included oxyhemoglobin saturation as measured by pulse oximetry, St George Respiratory Questionnaire Score, Medical Research Council dyspnea scale, body mass index, prealbumin level, arterial blood gas, rib cage range of motion, pulmonary function and the respiratory muscle strength.

**Table 2:** Linear regression analysis for changes in leg fatigue and dyspnea at the end of walking test

	Baseline, Mean (SD)	After 12 weeks, Mean (SD)	B (95% CI)^a^
Leg fatigue (Borg scale)			
Placebo (*n* = 32)	5.1 (1.9)	6.3 (2.2)	
Real (*n* = 30)	5.7 (3.3)	1.7 (1.6)	-4.8 (-5.8–-3.9)^b^
Dyspnea (Borg scale)			
Placebo (*n* = 32)	4.2 (2.7)	4.6 (2.8)	
Real (*n* = 30)	5.5 (2.8)	1.9 (1.5)	-3.6 (-4.3–-2.9)^c^

SD, standard deviation; B, unstandardized regression coefficient; CI, confidence interval; Placebo, placebo group; Real, real acupuncture group

^a^B (95% CI) indicates the estimated intervention effect of acupuncture against the placebo on changes in leg fatigue (or dyspnea) and its 95% confidence intervals

^b^Adjusted for the baseline values of leg fatigue

^c^Adjusted for the baseline values of dyspnea

### Mediation analysis

The results of the mediation analysis are shown in Table 3. In the simulation-based approach, when the changes in leg fatigue from baseline to postintervention were considered as the mediator, the TE of the real acupuncture against the placebo acupuncture regarding 6-min walk distance was 81.4 m (95% CI: 55.2–109.4), where the NDE (not mediated by changes in leg fatigue) was 47.1 m (4.6–85.2), the NIE (mediated) was 34.3 m (-2.1–82.1), and the PM was 42.1%. When the changes in dyspnea were considered as the mediator, the TE was 82.3 m (57.4–110.3), and the NDE, NIE, and PM were 9.8 m (-32.9–49.9), 72.5 m (31.3–121.0), and 88.1%, respectively. When the changes in leg fatigue and dyspnea were considered as the joint mediator, the TE, NDE, NIE, and PM were 82.1 m (56.7–112.6), -5.8 m (-55.4–43.9), 88.9 m (32.7–148.5), and 107.0%, respectively. The results of the two sensitivity analyses were similar to those of the main analysis.

**Table 3:** Causal mediation analysis

		Mediators	
	Leg fatigue	Dyspnea	Joint mediator
Simulation-based approach^a^			
TE, m (95% CI)	81.4 (55.2–109.4)	82.3 (57.4–110.3)	82.1 (56.7–112.6)
NDE, m (95% CI)	47.1 (4.6–85.2)	9.8 (-32.9–49.9)	-5.8 (-55.4–43.9)
NIE, m (95% CI)	34.3 (-2.1–82.1)	72.5 (31.3–121.0)	88.9 (32.7–148.5)
PM, %	42.1	88.1	107.0
Sensitivity analyses			
Addition of covariates^b^			
TE, m (95% CI)	82.8 (53.7–113.1)	83.3 (54.1–112.9)	84.1 (54.1–114.1)
NDE, m (95% CI)	49.4 (4.8–89.4)	11.1 (-34.7–53.9)	-5.2 (-59.3–45.5)
NIE, m (95% CI)	33.5 (-3.7–81.8)	72.2 (25.4–123.6)	89.3 (33.7–152.9)
PM, %	40.4	86.7	106.2
Weighting approach^a^			
TE, m (95% CI)	80.8 (58.1–105.3)	80.8 (58.1–105.3)	80.8 (58.1–105.3)
NDE, m (95% CI)	50.8 (6.7–89.1)	9.5 (-35.8–49.9)	-7.0 (-58.7–44.0)
NIE, m (95% CI)	29.9 (-6.1–75.5)	71.3 (30.4–120.8)	87.8 (31.5–146.9)
PM, %	37.1	88.3	108.6

TE, total effect; NDE, natural direct effect; NIE, natural indirect effect; PM, proportion mediated; CI, confidence interval

The results of the causal mediation analysis for each mediator are shown for the simulation-based approach as the main analysis and the two sensitivity analyses

TE is the estimated intervention effect of acupuncture against the placebo on 6-minute walk distance, NDE is the estimated effect of intervention on 6-minute walk distance not mediated by mediators, and NIE is the estimated effect of intervention mediated by mediators. Therefore, TE is the sum of NDE and NIE. PM is the proportion of TE accounted for by NIE, calculated as NIE divided by TE

^a^Adjusted for baseline values of 6-minute walk distance, leg fatigue, and dyspnea. ^b^Adjusted for baseline values of 6-minute walk distance, leg fatigue, dyspnea, age, body mass index and inspiratory capacity

## Discussion

To our knowledge, this is the first study to examine how much of the acupuncture-induced improvement in exercise capacity in patients with COPD is mediated by alleviation of leg fatigue and dyspnea. We found that acupuncture had significant therapeutic effects regarding leg fatigue and dyspnea compared to the placebo, and that about 40% of the total effect of acupuncture on exercise capacity was explained by leg fatigue alleviation, about 90% was explained by dyspnea alleviation, and almost 100% was explained by the two together. Studies investigating the main symptoms limiting exercise of patients with COPD in the case of walking load have descriptively reported that patients stop exercising due to dyspnea (69–77% of patients), leg fatigue (16–30%), or a combination of the two symptoms (1–12%) [4, 32, 33]. Although we cannot directly compare these reports since they did not investigate the mediating effects of leg fatigue and dyspnea, their findings do not contradict our findings.

The mediation effect increased significantly when changes in dyspnea were added as mediators. This indicates that the pathway that affects dyspnea without leg fatigue (path A-M2-Y in Fig. 1) plays a major role in the mechanism by which acupuncture improves exercise capacity. Improvement of respiratory muscle function and increased endogenous opioids may be involved in this pathway. Respiratory muscle dysfunction exacerbates dyspnea during exercise in patients with COPD by increasing perceived respiratory effort [34]. We observed in the previous clinical trial that the maximum inspiratory and expiratory mouth pressure and range of motion in the rib cage increased after acupuncture intervention [11], and that these changes possibly reflected the improvement of respiratory muscle function by acupuncture. These improvements may have resulted from acupuncture applied to acupuncture points located on the accessory respiratory muscles, which increased muscle blood flow [35] and reduced excessive muscle tension [36]. It has been reported that dyspnea in patients with COPD is modulated by endogenous opioids and improved by opioid treatment [37–39]. Acupuncture-induced increases in endogenous opioids may contribute to the reduction of dyspnea [40].

The point estimate for the natural indirect effect on exercise capacity due to changes in leg fatigue ranged from 29.9 m for the weighting approach to 34.3 m for the simulation-based approach. This value is comparable to the minimal important difference of 25–33 m for 6-minute walk distance in adults with chronic respiratory disease [18]. Various structural and functional abnormalities occur in the lower limb muscles of patients with COPD [41], and muscle deconditioning caused by them leads to the metabolite accumulation and acidosis associated with anaerobic metabolism, which occur at the early stage of exercise [42]. Therefore, some patients stop exercising due to leg fatigue rather than dyspnea [4, 32, 33]. Muscle fatigue in patients with COPD occurs in the dorsi and plantar flexors when a walking load such as the one in this study is applied [43]. Acupuncture stimulation to acupuncture points (ST 36, KI 3 in our study) on these easily fatigued muscles could improve oxygen utilization by increasing local muscle blood flow and oxygenation and lead to a reduction of leg fatigue and improved exercise capacity [35, 44–46]. However, the lower bound of the confidence interval of the indirect effect due to the change in leg fatigue was slightly below zero, so its mediation effect was not definite. Further studies are needed to elucidate how much of the total improvement of exercise capacity due to acupuncture is attained by the reduction of leg fatigue. In addition, the sum of the indirect effects when the two mediators were examined individually did not match with the indirect effect when they were considered jointly. This indicates the possibility that mediators affect one another [31]. As mentioned above, patients with COPD perceive leg fatigue through the activation of muscle afferents caused by the metabolite accumulation due to muscle deconditioning at the early stage of exercise [42]. These afferent signals increase the ventilatory response and dyspnea [20, 21]. In addition, it has been reported that blocking afferent signals from the lower limb muscles by spinal anesthesia can improve dyspnea and exercise capacity in patients with COPD [22]. Considering these previous reports that investigated the effects of leg fatigue on dyspnea and exercise capacity, as we hypothesized, there could be a pathway in the acupuncture mechanism that exercise capacity is improved because the changes in leg fatigue affect dyspnea (path A-M1-M2-Y in Fig. 1).

The strength of this study is that our analysis was based on placebo-controlled trial data. There was no psychological effect on the observed acupuncture mediation effects, and it should be interpreted as the mechanism of acupuncture. There are some limitations to the current study. First, there are four assumptions that need to be met for the identification of the natural direct and indirect effects; that is, there is no unmeasured confounding in (i) the exposure-outcome relationship, (ii) the mediator-outcome relationship, (iii) or the exposure-mediator relationship. And the fourth assumption is that there are no effects of exposure that confound the mediator-outcome relationship [24]. In this study, exposure refers to acupuncture or placebo intervention, mediators refer to changes in leg fatigue and/or dyspnea, and outcome refers to changes in 6-minute walk distance. We confirmed robustness on the addition of some covariates by sensitivity analysis, but the number of covariates that could be considered was limited by the sample size. Therefore, the possibility of unmeasured confounding cannot be ruled out. In addition, it cannot be denied that the existence of time-varying confounders affected by treatment may violate the fourth assumption. Second, our analysis was based on mediators and outcome measurements evaluated at two time points, but the real relationship can be more complicated because these variables can change over time while influencing each other. Third, the small sample size or data variability might have led to wide confidence intervals for all effects. Recently, a method that can estimate the analogue of natural direct and indirect effects in the presence of a time-varying confounder has been proposed, and has been extended to the setting of multiple mediators [47, 48]. By applying this method to large sample size data measured multiple times, a more detailed and reliable mediation effect of acupuncture could be evaluated. Finally, the exercise capacity we investigated for mediation effects was limited to walking loads only. It has been reported that the symptoms that limit exercise in patients with COPD differ between walking and cycling [4, 33]. Different exercise load settings may change the extent of leg fatigue and dyspnea’s contribution to the effect of acupuncture on exercise capacity. Therefore, one should be cautious when extrapolating the results to the exercise capacity measured with the different exercise types.

## Conclusion

This study showed that acupuncture improved exercise capacity (6-minute walk distance) in patients with COPD by alleviating leg fatigue and dyspnea (Borg scale). When performing acupuncture to improve exercise capacity, practitioners will likely find greater therapeutic benefits by focusing on reducing dyspnea and leg fatigue.

## Data Availability

The dataset supporting the conclusions of this article is available from the corresponding author, TK, upon request.

## Abbreviations

COPD: Chronic obstructive pulmonary disease
GOLD: Global Initiative for Chronic Obstructive Lung Disease
CI: Confidence interval
TE: Total effect
NDE: Natural direct effect
NIE: Natural indirect effect
PM: Proportion mediated
